# Differences in gait patterns, pain, function and quality of life between males and females with knee osteoarthritis: a clinical trial

**DOI:** 10.1186/1471-2474-10-127

**Published:** 2009-10-13

**Authors:** Ronen Debi, Amit Mor, Ofer Segal, Ganit Segal, Eytan Debbi, Gabriel Agar, Nahum Halperin, Amir Haim, Avi Elbaz

**Affiliations:** 1 Department of Orthopedics, Assaf Harofeh Medical Center, Zerifin, Israel; 2 APOS Research Group, APOS Therapy Center, Herzliya, Israel; 3 Department of Orthopedics, Tel Aviv Sourasky Medical Center, Tel Aviv, Israel

## Abstract

**Background:**

The aim of this study was to gain a deeper understanding of the gender differences in knee osteoarthritis (OA) by evaluating the differences in gait spatio-temporal parameters and the differences in pain, quality of life and function between males and females suffering from knee OA.

**Methods:**

49 males and 85 females suffering from bilateral medial compartment knee OA participated in this study. Each patient underwent a computerized gait test and completed the WOMAC questionnaire and the SF-36 health survey. Independent t-tests were performed to examine the differences between males and females in age, BMI, spatio-temporal parameters, the WOMAC questionnaire and the SF-36 health survey.

**Results:**

Males and females had different gait patterns. Although males and females walked at the same walking speed, cadence and step length, they presented significant differences in the gait cycle phases. Males walked with a smaller stance and double limb support, and with a larger swing and single limb support compared to females. In addition, males walked with a greater toe out angle compared to females. While significant differences were not found in the WOMAC subscales, females consistently reported higher levels of pain and disability.

**Conclusion:**

The spatio-temporal differences between genders may suggest underlying differences in the gait strategies adopted by males and females in order to reduce pain and cope with the loads acting on their affected joints, two key aspects of knee OA. These gender effects should therefore be taken into consideration when evaluating patients with knee OA.

**Trial Registration:**

The study is registered in the NIH clinical trial registration, protocol No. NCT00599729.

## Background

Osteoarthritis (OA) is the most prevalent form of arthritis in the elderly. Studies have shown that symptomatic knee OA is more prevalent in women than in men [1-3]. On the other hand, in some countries the gender difference in the prevalence of symptomatic knee OA is low or non-existent [4]. Radiographic knee OA, however, is much more prevalent in women than in men in comparison to symptomatic knee OA. For example, in the United States the prevalence of radiographic knee OA in adults ages 60 and older is 42.1% in women and 31.2% in men [4]. In Japanese patients 60-69 years of age the prevalence of radiographic knee OA is 57.1% in women and 35.2% in men [5]. This is not surprising considering that females have a higher risk of developing knee OA and functional disabilities compared to males [6-8]. OA is particularly disabling in weight bearing joints, such as the knees and hips. Ultimately, pain, stiffness and decreased range of motion lead to a loss of functional independence in daily tasks such as rising from a chair, climbing stairs and walking [9].

Males and females with knee OA have different gait patterns that are expressed in kinematic and kinetic parameters. Gender differences exist in the knee flexion angle, in the knee external moments (sagital, frontal and transverse plane) and in the knee internal moments (sagital and transverse plane) [10,11]. Concerning spatial and temporal aspects, McKean et al. found that while both males and females walk at the same self-selected speed and have the same stance time, males walk with a greater stride length in comparison to females [11].

Reviewing the gender differences in the healthy population reveals conflicting findings regarding the kinetic and kinematic parameters of gait. Some studies indicate that there are no gender differences in knee joint kinetics [12,13], while another study reports different kinematics between genders during gait [14]. The literature is also unclear regarding the differences in the stride characteristics between healthy genders. Some studies declare that there are no gender differences in the stride characteristics during walking [15,16]. On the other hand, some report that males and females walk at the same walking speed, but that females walk with a shorter step length [17,18], and some conclude that females walk more slowly than males and have a shorter step length [19,20]. Assuming that there are some gender differences in gait characteristics, it is important to understand these differences among patients with knee OA as they may explain the higher rate of knee OA in females compared to males.

There is still insufficient data regarding gender differences in certain spatio-temporal parameters that may be clinically relevant. Information on single limb support (SLS) (% Gait Cycle), for example, is scarce, yet this parameter may differ between genders. This is an important parameter because it represents the ability of the patient to bear single loads on the affected joint. Therefore, a further and deeper understanding of the differences between genders in SLS and other spatio-temporal parameters may help elucidate additional differences in gait patterns between males and females with knee OA.

Pain is a major symptom of knee OA and although gender differences in pain experience have been previously examined, results remain unclear. Some studies indicate that females report more severe clinical pain than males, while other studies have not found differences in pain levels between genders [21-24]. In addition, it is also known that patients with knee OA appraise their quality of life as lower compared to healthy age-matched individuals [25,26]. While some studies have shown that females with chronic pain feel more depressed than males [24,27], to our knowledge there is no data on the differences in quality of life perception between genders with knee OA.

The purpose of this study was to further examine the gender differences in patients with knee OA by evaluating the differences in gait spatio-temporal parameters, pain, quality of life, and function between males and females with knee OA. We hypothesized that males and females will present differences in some of the spatio-temporal parameters. Furthermore, if differences are found in the SLS phase we believe that differences will also be found in the level of pain (lower SLS values will correspond with higher pain levels, and vice versa).

## Methods

### Study participants

This study was approved by the Institutional Helsinki Committee Registry (Helsinki registration number 185/07, NIH protocol No. NCT00599729). All patients gave written informed consent before entering the study. Patients were recruited from the Orthopedics Outpatient Clinic of Assaf Harofeh Medical Center in Zerifin, Israel, and from the APOS Therapy Center in Herzliya, Israel. Patient eligibility was defined as symptomatic bilateral knee OA in the medial compartment for at least six months, fulfillment of the American College of Rheumatology (ACR) clinical criteria for OA of the knee [28], and radiographically assessed OA of the knee according to the Kellgren and Lawrence (K&L) scale [29].

Exclusion criteria were acute septic arthritis, corticosteroid injection within 3 months of the study, avascular necrosis, inflammatory arthritis, history of knee buckling, recent knee injury, neuropathic arthropathy, increased tendency to fall, lack of physical or mental ability to perform or comply with the study procedure, a history of pathological osteoporotic fractures, spinal or vascular claudication, and symptomatic degenerative arthritis in lower limb joints other than the knees. All patients underwent a gross motor function measure (GMFM) conducted by the senior orthopedic surgeon. All participants were instructed to refrain from taking pain medication, including paracetamol and NSAID's, for a period of 3 days prior to the examination.

### Protocol

All patients underwent a physical examination and most underwent a radiographic evaluation by the senior author (N.H.). The radiographs were obtained using a standardized technique [30]. Briefly, the images were 45 degree posteroanterior flexion weight-bearing radiographs. Patients stood with their weight equally distributed on the two extremities and with both knees flexed to 45 degrees. Toes were pointed straight ahead and the patellae touched the film cassette. The radiograph machine was positioned 101.6 cm away from the cassette. Measurements of height, weight and leg length (measured from the tip of the greater trochanter to the floor through the lateral melleolus in an upright standing position) were also collected [31].

All patients were required to walk barefoot at a self-selected speed on a computerized mat (GAITRite^® ^system, CIR Systems Inc. Peekskill, NY, USA). The reliability and validity of the computerized mat have been previously reported to be good to excellent [32,33]. Patients walked three meters before and after the walkway mat to allow sufficient acceleration and deceleration time outside the measurement area. Patients walked 6 times on the computerized mat and the mean value of the 6 walks was calculated for each parameter. Following the gait test, patients completed the Western Ontario and McMaster Universities Osteoarthritis Index (WOMAC) questionnaire and the SF-36 health survey.

The following spatio-temporal parameters were evaluated: absolute velocity (m/s), normalized velocity (m/s/leg length), cadence (steps/min), step length (m), normalized step length (m/leg length), swing phase (% gait cycle), stance phase (% gait cycle), single limb support phase (SLS) (% gait cycle), double limb support phase (DLS) (% gait cycle), base of support (m), and foot placement angle (deg).

The WOMAC questionnaire was divided into three categories: pain, stiffness and function. The SF-36 health survey was divided into 8 subcategories: physical functioning, role limitation due to physical health, role limitation due to emotional health, energy, emotional well being, social functioning, pain and general health.

### Statistical analysis

Data were analyzed with SPSS software version 14.0. The sample size was defined according to a power calculation that tested (2-tailed) the null hypothesis that the two population means were equal using the independent t-test. The study will have power of at least 80% to yield a statistically significant result. For SLS the minimal relevant difference was 1.5 with a standard deviation of 2.0 and 3.0 for males and females, respectively.

Independent t-tests were performed to compare males with females for continuous variables: age, BMI, spatio-temporal parameters, the WOMAC questionnaire and the SF-36 health survey. The chi-square test was calculated for the relationship between K&L grade and gender. Spearman correlations were calculated to find linear relationships between single limb support, WOMAC-pain, WOMAC-function and SF-36 health survey. Multiple bar graphs demonstrated the persistent differences between genders in ordinal level of age and BMI, following kruskal-Wallis nonparametric tests. Level of significance was set at *P *≤ 0.05, and was two-tailed.

## Results

134 patients suffering from bilateral knee OA of the medial tibiofemoral compartment participated in this study, 49 males (36%) and 85 females (64%). Since knee OA is more prevalent in females than in males [1-3] these figures were found acceptable. There were no significant differences in age, height, weight, BMI and K&L grading scale between genders. Normal GMFM was found in all patients.

9 females and 7 males did not comply with the required radiographic evaluation during the course of the study. Their grading was therefore excluded from the radiographic comparison. The radiographic comparison was carried out only to characterize the study population. We categorized the missing data as missing at random (MAR) and therefore assumed that the distribution with these patients included would remain the same. Furthermore the focus of our study was on the functional evaluation of knee OA. As such, the radiographic data, which is by nature static, did not affect the interpretation of the results. For these reasons we chose not to take new radiographs. Patient characteristics are presented in Table 1.

**Table 1:** Comparison of patient characteristics (mean (SD))

	**Males**	**Females**	P*
	**(n = 49)**	**(n = 85)**	
Age	66.9 (12.3)	67.5 (9.8)	0.7

Height (m)	1.68 (0.7)	1.54 (1.8)	<0.001

Weight (kg)	87.2 (15.5)	75.6 (15.5)	<0.001

BMI (kg/m^2^)	30.6 (5.1)	31.8 (5.8)	0.2

K&L Grade 1	8	13	

K&L Grade 2	8	17	

K&L Grade 3	11	24	

K&L Grade 4	13	22	0.93

* *P *≤ 0.05.

Independent t-tests were performed to examine gender differences in age, height, weight and BMI. A Chi-square test was calculated for the relationship between K&L grade and gender. The latter test covers the entire distribution of K&L scores.

BMI - Body Mass Index

K&L - Kellgren and Lawrence radiographic grading scale

Gait velocity and step length were normalized to leg length to eliminate the effect of the leg length differences that were found between genders. No differences were found in normalized velocity, normalized step length and cadence between genders. Significant differences were found in the gait cycle phases: stance, swing, SLS, DLS and in the foot placement angle (Table 2).

**Table 2:** Gender differences in gait parameters (mean (SD))

		**Males**	**Females**	P*
Velocity (m/s)		0.99 (0.19)	0.87 (0.24)	<0.001

Normalized Velocity (m/s/leg length)		0.11 (0.02)	0.10 (0.03)	0.52

Cadence (steps/Min)		105.4 (9.45)	102.8 (14.7)	0.25

Normalized Step Length (m/leg length)	Left	0.06 (0.009)	0.06 (0.008)	0.96
	
	Right	0.06 (0.008)	0.063 (0.01)	0.33

Swing (% Gait Cycle)	Left	37.7 (2.1)	36.1 (3.4)	0.001
	
	Right	37.0 (2.8)	35.9 (3.8)	0.05

Stance (% Gait Cycle)	Left	62.2 (2.1)	63.9 (3.4)	0.001
	
	Right	63.0 (2.8)	64.1 (3.8)	0.05

Single Limb Support (% Gait Cycle)	Left	37.0 (2.8)	35.8 (3.8)	0.03
	
	Right	37.7 (2.1)	36.2 (3.4)	0.003

Double Limb Support (% Gait Cycle)	Left	25.3 (3.9)	28.0 (6.7)	0.004
	
	Right	25.4 (3.9)	28.2 (6.8)	0.004

Toe Out Angle (Deg)	Left	8.5 (6.0)	5.4 (5.3)	0.003
	
	Right	9.9 (5.2)	6.8 (5.2)	0.001

* P ≤ 0.05.

Independent t-tests were performed to compare males with females for continuous variables. The left and right limbs values included in the table represent the more affected limb of the patients.

No significant differences between genders were found in the WOMAC pain, stiffness and function categories or in the overall score. Nevertheless, the mean score for each of the WOMAC categories and the overall score were higher in females compared to males and the p-values for the categories were all close to the significance threshold (*P *≤ 0.05) (Table 3).

**Table 3:** Gender differences in the level of pain and quality of life (mean (SD))

	**Males**	**Females**	P*
**WOMAC Categories**			

WOMAC - Pain (VAS Scale - cm)	4.0 (2.4)	4.6 (2.4)	0.12

WOMAC - Stiffness (VAS Scale - cm)	3.4 (3.1)	5.0 (4.3)	0.12

WOMAC - Function (VAS Scale - cm)	4.0 (2.6)	4.8 (2.6)	0.11

WOMAC Final Score (VAS Scale - cm)	3.98 (2.4)	4.78 (2.52)	0.07

**SF-36 Categories**			

Physical Functioning	47.75 (24.32)	42.28 (25.16)	0.22

Role Limitation Due to Physical Health	52.04 (40.45)	37.95 (38.72)	0.04

Role Limitation Due to Emotional Health	62.58 (42.29)	55.82 (44.20)	0.39

Energy	61.53 (18.60)	49.93 (23.90)	0.004

Emotional Well Being	74.04 (17.79)	66.93 (21.00)	0.04

Social Functioning	73.46 (26.22)	67.62 (28.69)	0.24

Pain	47.60 (26.76)	42.01 (25.02)	0.23

General Health	66.14 (18.21)	57.71 (19.61)	0.01

*P ≤ 0.05

WOMAC - 24 questions in a format of VAS scale. Five questions representing pain, two questions representing stiffness and 17 questions representing function. Lower scores indicate better conditions.

SF-36 quality of life health survey - the score range is between 0-100. Higher scores indicate a better condition.

Significant differences were found in the following SF-36 subcategories: Role limitation due to physical health, energy, emotional well being and general health. Males consistently reported significantly higher values compared to females in the above categories. While the subcategories of physical function, role limitation due to emotional health, social functioning and pain were not significantly different between genders, male reported higher values in all these categories (Table 3).

**Figure 1:**
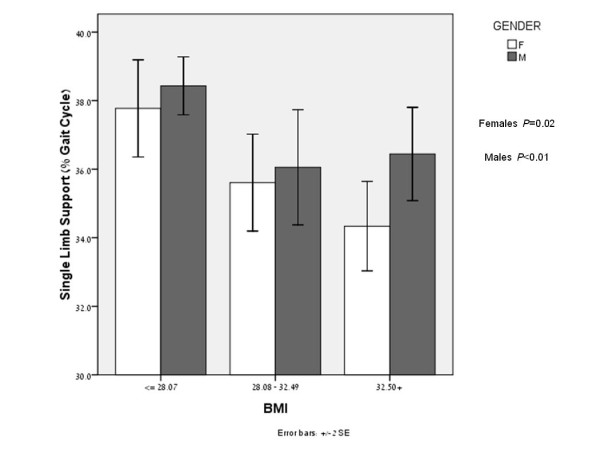
**SLS values of males and females, after dividing BMI into tertiles**. Significant difference were found in the BMI tertiles (*P *< 0.01 and *P *= 0.02 for males and females, respectively). SLS values are consistently lower in females. Females and males with higher BMI values have greater difficulty maintaining single limb loads. This is illustrated by the decreased SLS values in both genders as BMI increases.

**Figure 2:**
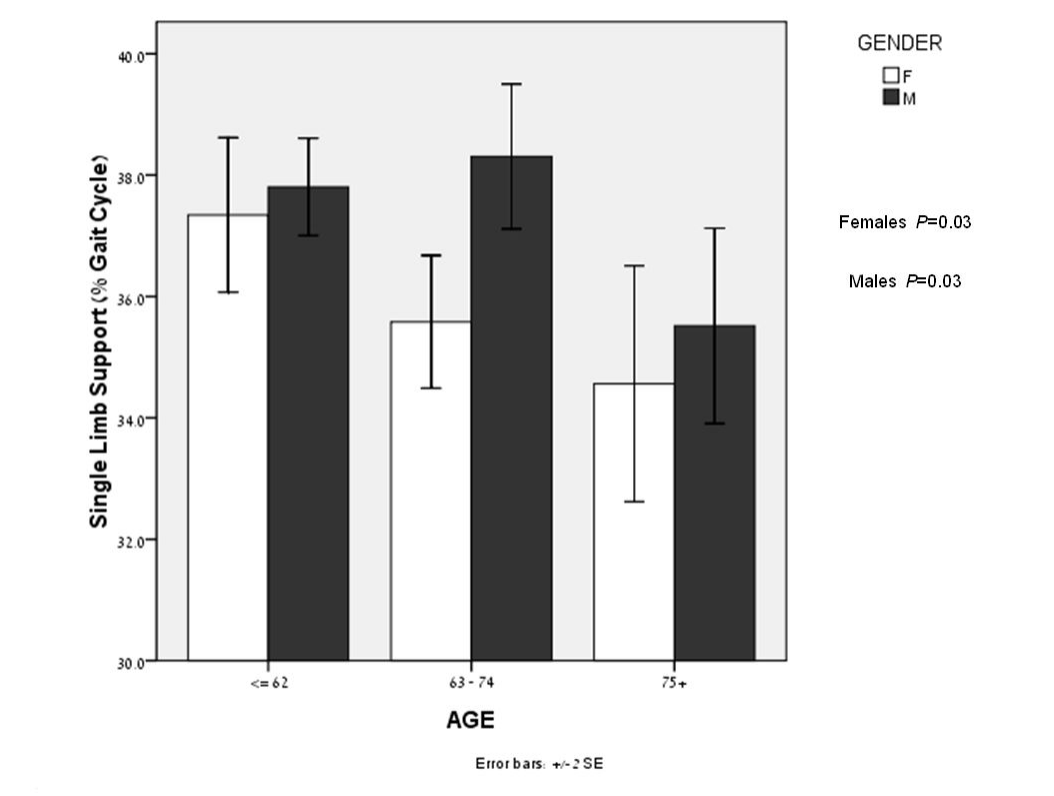
**SLS values of males and females, after dividing age into tertiles**. Significant difference were found in the age tertiles (*P *= 0.03 and *P *= 0.03 for males and females, respectively). SLS values are consistently lower in females. Older females and males have greater difficulty maintaining single limb loads. This is illustrated by the decreased SLS values in both genders as age increases.

A further examination of the differences between genders revealed a significant difference in the SLS mean value between genders after dividing males and females into tertiles according to their BMI (females and males; *P *< 0.01 and *P *= 0.02 respectively) and age (females and males; *P *= 0.03 and *P *= 0.03, respectively). This analysis was conducted in order to further examine and understand the changes in SLS according to the tertile distribution of age and BMI, two parameters that are known to correlate well with knee OA severity [34,35]. Figures 1 and 2 illustrate a consistent difference in SLS phase between genders in the tertile distribution of age and BMI. The SLS values shown are for the left limb, although similar results were seen for the right limb. The correlations between the SLS parameter and the WOMAC-pain, WOMAC-function and SF-36 quality of life subcategories are presented in Table 4.

**Table 4:** Correlations between SLS and WOMAC-pain, WOMAC-function and quality of life

**Parameter**	**Single Limb Support - r**		P*	P*
	**Females**	**Males**	**Females**	**Males**
WOMAC - Pain	0.46	0.5	<0.001	<0.001

WOMAC - Function	0.47	0.55	<0.001	<0.001

SF-36	0.49	0.59	<0.001	<0.001

* *P *≤ 0.01

## Discussion

One of the purposes of this study was to add new information regarding the gait spatio-temporal parameters of males and females with knee OA. We found that males and females did not differ in the parameters of normalized velocity, normalized step length and cadence. These findings are in partial agreement with the findings of a previous study by McKean et al. [11]. McKean et al. found no gender differences in walking speed, but found gender difference in step length while our study did not. A possible explanation for this discrepancy is that in the study of McKean et al. males were significantly taller than females, yet the data was not normalized, as it was in our study, to eliminate the effects of height on the data. A study by Kerrigan et al. reported that healthy males who walked at the same walking speed as females demonstrated lower cadence and longer step length than did the females [14]. In the current study we found that males and females with knee OA walked at the same walking speed and had similar cadence and step length.

A possible explanation for these changes might relate to the nature of OA disease. It is possible that by reducing their step length and increasing their cadence, males were able to reduce the impact loading from their affected limb. This is physically plausible since decreasing step length causes a decrease in the vertical ground reaction forces (assuming no change in gait velocity) [36]. This might be a general strategy adopted by males in order to reduce loads from their affected joints.

The results of the current study demonstrate gender differences in several spatio-temporal parameters, indicating a difference in gait patterns between genders with knee OA that correlates with the differences in the level of pain, function, and quality of life. Although normalized gait velocity, normalized step length and cadence did not differ between males and females, significant differences were found in the gait cycle phases: stance, swing, SLS and DLS. Males had a smaller stance and DLS, and a larger swing and SLS compared to females.

SLS (a % of gait cycle) expresses a unique phase in the gait cycle when the body weight is entirely supported by one limb while the contralateral limb swings forward. In the healthy population, this phase accounts for 38-40% of the gait cycle [37,38]. A previous study showed that there are lower SLS values in both limbs among patients with knee OA compared to the SLS values of healthy individuals [39].

We hypothesized that the difference in SLS between genders in the current study is a result of different perceptions of pain. We assumed that higher pain would lead females to avoid supporting their entire body weight on the affected limb. This would therefore decrease their SLS and increase their DLS. To examine this hypothesis we calculated the correlation between the SLS phase and the level of pain, function and quality of life. We found moderate correlations for all parameters with no significant differences between genders. While our results showed no significant difference in the WOMAC pain scores between males and females, females always reported higher levels of pain compared to males. Future studies should examine the differences in pain between genders in greater depth in order to determine if a true difference exists.

We also thought that the difference in SLS may be due to gender differences in body mass index and radiographically assessed OA severity levels (K&L). This hypothesis was eventually rejected since no such differences were found between genders in this study.

We further examined the SLS differences in BMI and age tertiles, which are two parameters that correlate with knee OA severity. It was found that that BMI and age are inversely related to SLS value.

Males and females in the current study presented different foot placement angles during walking. This was an interesting finding since this parameter is particularly relevant to patients suffering from knee OA. A previous study analyzed the relationship between the toe out angle parameter during gait and knee OA [40]. Walking with a higher toe out angle shifts the ground reaction force vector closer to the center of the knee joint, thus decreasing the moment arm acting to adduct the knee joint. Theoretically, this should help decrease loads on a joint affected by knee OA [41-43]. In the current study males walked with a greater toe out angle than females. Although this study did not measure the forces and moments acting on the knee joint, males may have adopted a greater toe out angle as a strategy of decreasing loads from the affected compartment. Another method of decreasing loads on a joint is to reduce SLS. This strategy may have been adopted by females since they demonstrated lower SLS values compared to males.

These results suggest that different methods of evaluation may be used in either male or female patients. With regard to male patients, the toe out angle gait parameter may be used as a measure of the functional severity of knee OA. On the other hand, clinicians may be able to use the SLS gait parameter to measure the functional severity of knee OA in female patients. Future studies should examine the gender differences in SLS and foot placement angle to help elucidate the unique relationship between these parameters and the forces and moments acting on the body.

The data from the self-reported questionnaires revealed differences between genders in the perception of pain, function and quality of life. Females reported a significantly poorer quality of life compared to males and probably had higher levels of pain and disability compared to males. A study by Tsai found that although females reported higher levels of pain intensity and had a greater depressive tendency compared to males, the pain intensity during walking did not differ between genders [44]. In addition, a study by Rollnik et al. showed that females with chronic pain were more depressed than males [27]. Overall, these studies and the current study suggest that females may experience worse symptoms of knee OA compared to males. Interestingly, our results showed that males and females did not differ in their radiographic assessment of OA severity. This paradox supports numerous studies that questioned the correlation between the radiographic assessment and the functional condition of a patient with knee OA [45,46].

This discrepancy highlights the importance of a comprehensive evaluation of a patient with knee OA using a variety of assessment tools, especially objective functional parameters that are able to reveal gender differences. It would be logical to include reported level of pain and function (WOMAC) and spatio-temporal parameters measured in a gait analysis test, specifically SLS and foot placement angle, as these are objective functional tools that were able to find gender difference in knee OA in this study.

This study had some limitations that withhold more established conclusions regarding the study's findings. First, a kinetic analysis of the patients during gait was not carried out. Integrating the current study findings on the gender differences in both SLS and toe out angle with kinetic data would have provided better information regarding the external adduction forces acting on the knee joint. Second, the studied population was limited to patients suffering from bilateral knee OA of the medial compartment for at least 6 months. Information regarding the specific length of time that the patients had been suffering from OA is important, since this may have influenced the results. We recommend that future studies incorporate this time factor into the study design. We also recommend an extensive examination of the correlation between the SLS phase and the toe out angle using a kinetic analysis of patients with knee OA.

## Conclusion

There are significant gender differences in most of the spatio-temporal gait parameters of patients with knee OA, specifically in all of the gait cycle phases (stance, swing, DLS and SLS) and in the foot placement angle. Some of these differences suggest that males and females adopt different gait strategies in response to OA disease. When evaluating patients with knee OA these gender effects should be taken into consideration. We also found that females have a poorer quality of life perception than males. Our results highlight the importance of using a variety of tools, especially objective functional parameters, when evaluating knee OA severity. More research is recommended on the gender differences in SLS, toe out angle, pain, function and kinetic parameters.

## Competing interests

The authors declare that they have no competing interests.

## Authors' contributions

RD conceived of the study, participated in its design, revised the manuscript and gave final approval. AM conceived of the study, participated in its design, revised the manuscript and gave final approval. OS carried out data collection, drafted the manuscript and gave final approval. GS carried out data collection, drafted the manuscript and gave final approval. ED carried out data collection, drafted the manuscript and gave final approval. GA revised the manuscript and gave final approval. NH revised the manuscript and gave final approval. AH revised the manuscript and gave final approval. AE conceived of the study, participated in its design, revised the manuscript and gave final approval. All authors read and approved the final manuscript.

## Pre-publication history

The pre-publication history for this paper can be accessed here:


